# Herbal medicines use and associated factors among pregnant women in Debre Tabor town, north West Ethiopia: a mixed method approach

**DOI:** 10.1186/s12906-021-03439-3

**Published:** 2021-10-26

**Authors:** Getu Tesfaw Addis, Birhanu Demeke Workneh, Mesfin Haile Kahissay

**Affiliations:** 1 Department of Pharmacy, College of Medicine and Health Sciences, Debre Tabor University, Debra Tabor, Ethiopia; 2 Department of Pharmacy, College of Medicine & Health Sciences, Wollo University, Dessie, Ethiopia

## Abstract

**Background:**

Use of herbal medicines during pregnancy has been increase in many developing and developed countries. In spite of the studies done on herbal medicine, no study has addressed use of herbal medicine among pregnant women in Debre Tabor Town. Hence, the major aim of this study was to assess the prevalence of herbal medicine use and associated factors.

**Methods:**

A community based explanatory sequential mixed methods was employed. The quantitative method used cross-sectional study design with a sample size of 267 women, also 12 participants in a group for focus group discussion and 6 in-depth interviews from focus group were included for a qualitative part using a purposive sampling technique. The data were coded and entered into Epidata 4.2.0.0 and analysis was done using SPSS version 25, while thematic analysis was used for qualitative data. Bivariate and multivariate logistic analyses were used to assess associations between dependent and independent variables.

**Results:**

Ninety-five (36.3%) of pregnant women used herbal medicine during pregnancy. Prior use of herbal medicine (AOR: 3.138; 95% CI: 1.375, 7.162), unable to read & write (AOR: 9.316; 95% CI: 2.339, 37.101), presence of health problems (AOR: 3.263; 95% CI: 1.502, 7.090), drug availability (AOR: 9.872; 95% CI: 4.322, 22.551) and distance to the health facilities (AOR 6.153; 95% CI 2.487, 15.226) were significantly associated with use of herbal medicine. Only 5(5.3%) of herbal medicine users disclosed their herbal medicine use to their healthcare providers*. Zingiber officinale, Eucalyptus globulus, Rutachalepensis, Linumusitatissimum,* and *Moringa stenopetala* were the most commonly used herbal medicines by pregnant women.

**Conclusions:**

The use of herbal medicine during pregnancy is a common practice and significantly associated with educational status, prior use of herbal medicine, presence of health problems, drug availability and distance to the health facilities. Since there was high prevalence and low disclosure rate of herbal medicine use, it should be ensured that physicians/midwives establish a good level of communication with pregnant women.

**Supplementary Information:**

The online version contains supplementary material available at 10.1186/s12906-021-03439-3.

## Background

Traditional medicine is comprehensive in nature on the grounds that it delivers numerous issues identified with socio- cultural, economic and ecological context of a community [1]. For instance, one of the working definition of “traditional medicine” by World Health Organization is that: “it is the sum total of all knowledge and practices, whether explicable or not used in the diagnosis, prevention and elimination of physical, mental or social imbalances and relying exclusively on practical experience and observation handed down from generation to generation, whether orally or in writing [2]. Herbal medicines, which is part of traditional medicines are characterized as plant-determined material or preparations saw to have remedial advantages; they frequently contain crude or processed ingredients from at least one plants. Herbal medications incorporate herbs, home remedies, herbal preparations, and finished home-grown items that contain portions of plants or other plant materials as active ingredients and used by the general population as well as pregnant women [3].

Pregnancy is a condition associated with immense physiological alterations resulting in many pregnancy-related problems, including nausea, vomiting, constipation, and heartburn [4]. These ailments usually result in pregnant women self-medicating using traditional medicine, especially herbs [5]. Furthermore, pregnant women, in developing countries, use herbal medicines due to its easy accessibility, affordability, lack access to health care and belief that herbs are safer for the fetus than modern medicine because they are natural products [4]. The use of herbal medicines has increased in most countries in Africa and Asia as in many other developed countries. Approximately 65–80% of the world’s population use traditional medicine as their primary form of health care, including use during pregnancy [6]. In sub-Saharan Africa, up to 80% of the population uses TM to meet their health care needs, including use during pregnancy [7]. Like most African countries, Ethiopia relies heavily on indigenous medicine for its primary health care services [3]. Despite the increased consumption of herbal medicines among pregnant women all over the globe, majority of them are unaware of the potential side effects and a potential teratogenicity of some herbal products [8].

In Ethiopia, more than 80% of the population use traditional medicine. A study done in *Hossana* town, Southern Ethiopia showed that 73.1% of pregnant women use herbal medicines and the most common herbs used were ginger (55.8%), garlic (69.8%), and *tenaadam* (26.4%) [9]. Similar study conducted in *Nekemte* Hospital, western Ethiopia reported that the most commonly used herbs, by pregnant women, were ginger (44.36%) and Garlic (37.32%). Nausea (23.90%) and morning sickness (21.05%) were the most common reasons for herbal use in pregnancy [10]. Another study conducted in *Gondar, Ethiopia,* found ginger (40.7%) and garlic (19%) were the two most commonly used herbs in pregnancy. Common cold and inflammation were the most common reasons for herbal use [11].

Several studies have also reported the association between different socio-demographic characteristics of pregnant women, and utilization of herbal medicines during pregnancy. The most important variables listed by scholars includes: low level of education, rural residence, no occupation, older, and positive attitude towards the use of herbal medicines [9–12].

In spite of the studies done on herbal medicine, there is a limited data on the prevalence and correlates of herbal medicine use among pregnant women using mixed method approach. Therefore, the objective of this study was to assess the prevalence of herbal medicine use and associated factors among pregnant women in Debre tabor town, Northwest Ethiopia using mixed study design.

## Methods

### Study design and setting

**Fig. 1:**
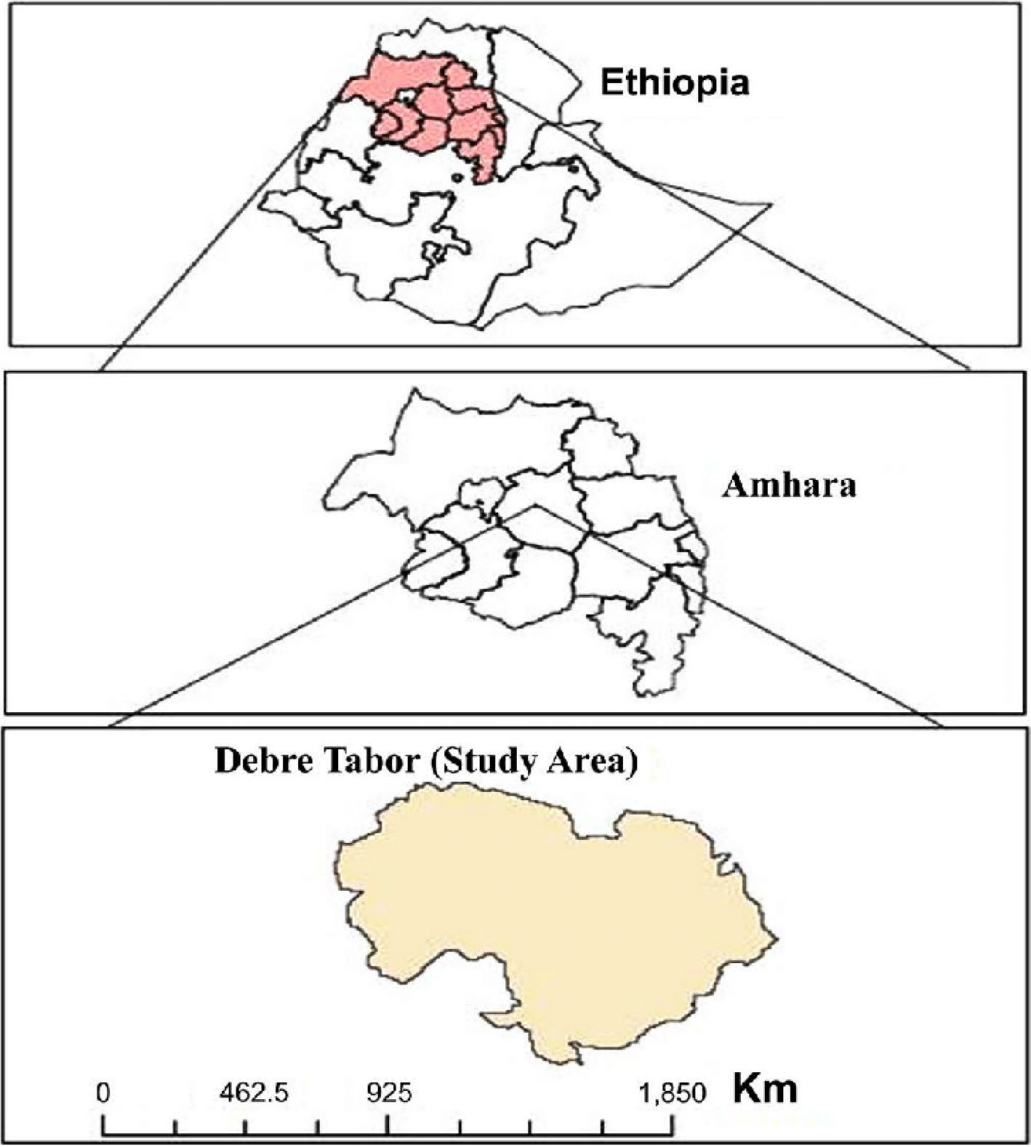
Map of the study area, Debre Tabor Town, South Gondar, Ethiopia

A Community based cross-sectional explanatory sequential mixed methods approach was conducted to assess the use of herbal medicines and associated factors among pregnant women in *Debre Tabor* Town from September 1 to 30, 2019. The town was selected as the study area because it has unique cultural, environmental landscape, and variety of plant species that do affect the pattern of herbal medicine uses. *Debre Tabor* town is located in south Gondar zone, northwest Ethiopia, 667 km away from Addis Ababa (the capital city of Ethiopia). Based on the information from *Debre Tabour* town Administrative Bureau, the town has 10 kebeles and with an estimated total population of 111,029 (Fig. 1).

### Conceptual framework

**Fig. 2:**
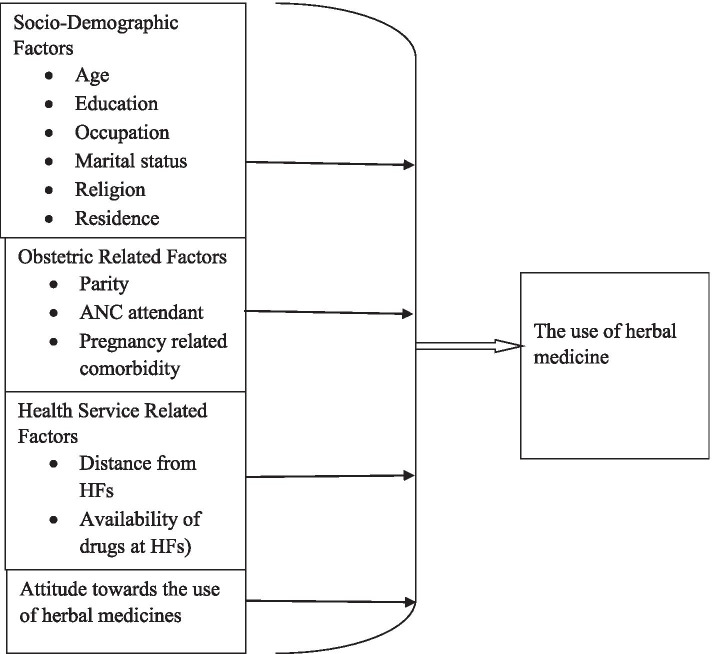
Conceptual framework of the use of herbal medicines and associated factors among pregnant women in *Debre Tabour* Town, 2019 [8, 10, 12, 15]

To understand factors that motivate the use of herbal medicine among the study community, a conceptual framework was adapted from literatures [9, 11, 13, 14]. It suggests that herbal medicine uses by pregnant women depends on four core components which includes: sociodemographic factor, obstetric related factors, health service-related factors and attitude towards the use of herbal medicines. This conceptual framework also effectively integrates different factors which may influence the use of herbal medicine by pregnant women (Fig. 2).

### Sample size determination and procedure

The source populations were all pregnant women residing in *Debre Tabor* town, while those pregnant women who were registered at the health extension workers registration book during the data collection period were taken as a study population. The sample size was calculated using a single population proportion formula [13] with the assumption of 95% confidence interval, 5% margin of error and 48.6% prevalence [11] of herbal medicine use among pregnant women and 10% for possible non response was taken to determine a final sample size of 267. By dividing the total number of pregnant of the ten *kebele* (650) with the total sample size (267), an interval of two was used to select household during the one-month data collection period by using systematic random sampling technique. The first household was selected using the lottery method. Then the next household was selected with an interval of two. If the study participant was not available at the first visit, this household was revisited once the same day or following day. If not available again, the study participant was considered as non-respondent. Pregnant women who were willing to share the information and available at the time of data collection were included in this study.

Qualitative Approach: The health extension workers also helped to select 12 participants for one focus group discussion (FGD) and six individuals were selected purposively for in-depth interviews from the FGD based on their knowledge about local herbs and ability to describe their experiences in the focus group.

### Data collection tools and techniques

Data collection was performed by three data collectors (BSC nurses) through interviewer-administered questionnaire. The data collectors were properly trained on the purpose of the study, the content of the questionnaire, interviewing techniques, how to approach the respondents, and securing their permission for interview prior to the data collection process. The data collection tool was adapted from different literature after review of the published studies [8, 10, 13–15] and prepared in English. This was translated to local language (*Amharic*) and then back translated to English in order to ensure consistency. The data collection instrument was pretested on 26 pregnant women who were not included in the final analysis and relevant modifications were done before the commencement of actual data collection. The final questionnaire constituted 33 items that were divided into five main parts (Annex). The first section includes questions about socio-demographic data, the second section includes questions about related to the use of herbal medicines, the third section includes questions about some obstetric related factors, the fourth section includes questions about accessibility of health facility and finally, the last section assessed the attitude of sample population regarding use of herbal medicine during pregnancy with five Likert-scale including seven questions. Each question had five choices (ranging from strongly agree to strongly disagree). Item analysis was done, and the internal consistency reliability had a Cronbach’s alpha of 0.82. The answer scores for each question choice and question in both groups were added up and their means and standard deviations calculated. The question scores ranged from 7 to 34 representing the most negative and the most positive attitude respectively. The higher the question score, the more positive the attitude is. After computing the median of all respondents’ responses, the median score of each respondent was dichotomized as have a positive attitude or negative attitude. A score of ≥19 was defined as a “positive attitude towards the use of herbal medicines during pregnancy,” and a score of < 19 was defined as “negative attitude towards the use of herbal medicines during pregnancy.”

Positive attitude ≥median.

Negative attitude <median.

In this study, respondents were considered as herbal medicine users if they have taken herbal medicine(s) through oral, intra-vaginal or topical routes during gestational period. Other preparations that are consumed as routine meal preparations and those that are taken as nutrients were excluded.

The qualitative data collection was conducted using an interview guide through probing questions by the principal investigator. Interviews lasted 30–90 min using an audio recorder as well as a filled note was taken by one trained note taker. Participants were briefed about the aim of the study by the principal investigator. Verbal informed consent was obtained from the participants and place of the interview was arranged between the principal investigator and the interviewee, the FGD and in-depth interviews were conducted at the public places in the villages.

### Data processing and analysis

Quantitative data was entered into EpiData version 4.2.0.0 and exported to Statistical Package for the Social Sciences (SPSS) software version 25.0 for analysis. Descriptive statistics (frequencies, percentages, mean, and standard deviation) and inferential statistics (bivariate and multivariate analyses) were calculated using bivariate and multivariate logistic regression with a 95% confidence interval (CI). Bivariate logistic regression was used to measure the association between independent variables and herbal medicine use. First bivariate logistic regression was performed to identify candidate variables for multiple logistic regressions. Those variables with a *p*-value of below 0.25 in the bivariate analysis were fitted to multiple logistic regressions. Model fitness was tested using the Hosmer and Lemeshow’s test and it was insignificant. Multi-collinearity was checked using variance inflation factor (VIF). Covariates with a p-value of below 0.05 in multivariate logistic regression were considered statistically significant factors with the dependent variable (herbal medicine use). Finally, the crude and adjusted odds ratio (OR) with 95% confidence interval (95% CI) were computed to measure the strength of the association between the outcome and the independent variables.

A focus group discussion and interviews were audio recorded and transcribed verbatim in Amharic. Texts were read independently by the PI and another professional who speaks the local language and codes were developed in reference to the research questions. Each of the codes were organized into higher-order conceptual themes. These individual codes and themes were discussed at group meetings until consensus was reached on basic themes and subthemes across a focus group and interviews. Finally, the themes were incorporated into a conceptual model of the participants and their use of herbal medicines and associated factors among pregnant women [13]. Sections of original transcripts and key quotes considered to be illustrative of the emerging themes were translated into English to facilitate discussion with the full research team. Data analysis was supported by the use of NVivo 10 computer software.

### Data quality assurance

The quality of the quantitative data was assured by pre-testing the questionnaire on 10% of the sample size (26 pregnant women) in a Town which is different from the study area prior to the start of the actual study to test the fitness of the questionnaire for the study settings. Training about the data collection tool as well as data collection procedures was given to data collectors and supervisors for a total of 1 day prior to the data collection process. The principal investigator was verifying the data during the data collection and every questionnaire was checked every day after data collection before data entry. Data was kept in the form of a file in a private secure place and confidentiality of respondents was ensured by not recording names or any personal identity.

The transcripts of qualitative data were shared with research participants to confirm the verbatim accurately reflected their experiences. The data was assured by an expert from the department of social and administrative pharmacy who confirmed the interpretations accurately. Moreover, a conceptual framework was used to guide the study, methodological triangulation (the data collected in the quantitative part and the qualitative part were compared and contrasted) and more than one investigator was involved in this study. Moreover, to ensure reliability of the qualitative tool or research team credibility, transferability, dependability and confirmability aspects of the research were taken into account [13].

### Issues of reflexivity: GT status as an insider

The first author’s (GT) “native” status offered both opportunities and limitations for the study. He approached this study as an “*Amharic*” speaker and tradition bearer, a member of the “*Amhara*” elite, and also as a senior pharmacy professional. He was able to use existing networks and contacts within the indigenous institutions and local health officials, thereby gaining access to a very wide cross-section of people. He carefully reflected on how the data collection process influenced his own perceptions, and how other people respond to him. He was also faced with the challenge of being perceived as a powerful individual due to his position as a member of the elite and a senior university lecturer. The use of open-ended questions, as well as informal conversations with informants on topics they themselves raised, were among the ways pursued to mitigate these challenges.

## Results

### Quantitative results

#### Socio-demographic characteristics of respondents

Out of 267 pregnant women invited to participate, 262 of them completed the survey giving a response rate of 98.2%. The age range of respondents was from 18 to 46 with a mean of 32.68 years (SD = ± 6.47 years). Of the total number of respondents, 178 (67.9%) of the study participants were a follower of Orthodox Christian followed by Muslims 66 (25.2%). In terms of educational level, 69 (26.3%) of respondents had completed secondary school. The socio-demographic and pregnancy related characteristics of respondents are summarized in Table 1.

**Table 1:** Socio demographic characteristics and factors associated with herbal medicine use among respondents, debre tabor, 2019 (*N* = 262)

Variables	HM use			
	Yes(n)	No(n)	COR(95%CI)	AOR(95%CI)
**Age group in year**				
15–19	1	4	1	1
20–29	18	72	1(.105–9.501)	1.708(.048–6.646)
30–39	54	70	.324(.035–2.983)	.210(.006–7.331)
40–49	22	21	.239(.025–2.313)*	.168(0.005–6.275)
**Educational status**				
Unable to read & write	17		7.353(2.760–19.589)***	9.316(2.339–37.101)*
Read & write	18	44	4.074(1.511–10.984)*	2.830(0.687–11.652)
Primary education	17	19	1.863(0.649–5.345)	1.064(0.239–4.744)
Secondary education	28	20	1.190(0.435–3.256)	0.678(0.164–2.806)
Diploma and above	15	9	1	1
**Residence**				
Peri urban Urban	57 38	98 69	.947(0.567–1.582) 1	1.265(.589–2.714) 1
**Previous use of HM**				
Yes	20	78	3.287(1.841–5.866)***	3.138(1.375–7.162)*
No	75	89	1	1
**Presence of health problems**				
Yes	37	105	2.655(1.581–4.458)***	3.263(1.502–7.090)**
No	58	62	1	1
**Drug availability in the HF**				
No	32	130	6.917(3.948–12.118)***	9.872(4.322–22.551)***
Yes	51	87	1	1
**Distance to HF**				
≥5 km	51	127	2.739(1.600–4.689)***	6.153(2.487–15.226)***
< 5 km	44	40	1	1
**Attitude towards the use of HMs**				
Positive attitude	34	100	2.678(1.590–4.510)***	2.840(1.248–6.464)
Negative attitude	61	67	1	1

Significant at *p*-value < 0.05*, *P*-value ≤0.005**, *p*-value ≤0.001***

#### Prevalence and reasons of herbal medicine use during pregnancy

**Fig. 3:**
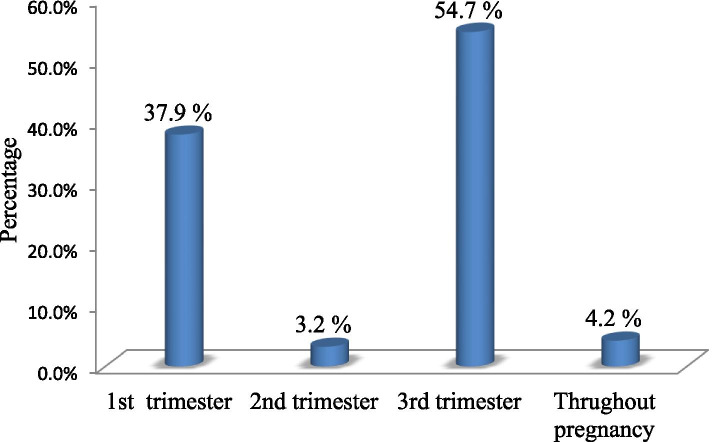
Use of Herbal Medicines in Different Trimesters in Pregnant Women at *Debre Tabor* town, 2019

**Fig. 4:**
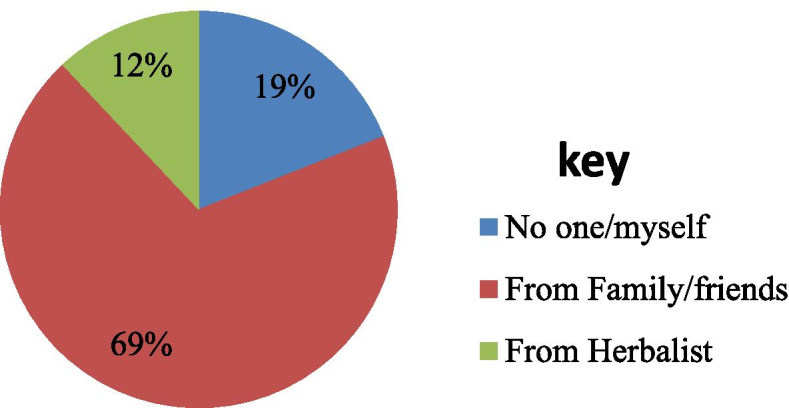
Source of information on herbal medicine for pregnant women at *Debre Tabor* Town, 2019

The prevalence of herbal medicine use among pregnant women in Debre Tabor Town was 95 (36.3%), with more than half of them (54.7%) used in the third trimester (Fig. 3). The most common reason for herbal medicines used during pregnancy was the ease of availability when they need them (*n* = 80, 84.2%). Similarly, the most common reason for not using herbal medicines during pregnancy among non-users was not properly processed (*n* = 119, 71.3%) (Table 2). Regarding respondent’s discussion with health care providers (HCPs) about HM use during pregnancy, the majority of the respondents (*n* = 90, 94.7%) didn’t disclose their use of herbal medicines with health care providers, only few of them (*n* = 5, 5.3%) discussed use of herbal medicines with doctors/midwives. The most common reason for the non-disclosure was doctors/midwives did not ask this query (*n* = 47, 52.2%). With regard to the source of information about the use of herbal medicines during pregnancy, most participants (69%) used herbal medicines based on advice from family/friends (Fig. 4).

**Table 2:** Prevalence and reason for use of herbal medicine among respondents, Debre Tabor Town, Ethiopia, 2019 (N = 262)

Variables	Frequency, n (%)	
**Herbal medicine use during current pregnancy(*N*** **= 262)**		
	Yes	95 (36.3)
	No	167 (63.7)
**Reason for use of herbal medicines (*N*** **= 95)**		
Believes in effectiveness of herbal medicines	Yes No	59(62.1) 36 (37.9)
They are safe to use during pregnancy	Yes No	17(17.9) 78(82.1)
It is part of our culture to use it	Yes No	54(56.8) 41(43.2)
It is always available when I need them	Yes No	80(84.2) 15(15.8)
To prevent miscarriages	Yes No	30 (31.6) 67(70.5)
**Reasons for nonuse of herbal medicines(*N*** **= 167)**		
The side effects could be dangerous	Yes No	95(56.9) 72(43.1)
It is not safe for pregnant women	Yes No	50(29.9) 117(70.1)
I don’t believe in the effectiveness of herbal medicines	Yes No	66 (39.5) 101(60.5)
It is not properly processed	Yes No	119 (71.3) 48(28.7)
Friends/family don’t advise me not to use it	Yes No	30 (18.0) 137(82.0)
Health professionals don’t advise me not to use it	Yes No	26 (15.6) 141(84.4)
**Discuss with HCPs about HM use (*N*** **= 95)**		
Yes		5(5.3)
No		90(94.7)
**Reason for non-disclosure (*N*** **= 90)**		
Forget to inform		29(32.2)
Doctors/midwives didn’t ask		47(52.2)
Afraid of doctors or midwives response		11(12.2)
It was not important to disclose/talk		3(3.3)

#### Factors associated with the use of herbal medicines during pregnancy

Results of the bivariate analysis showed that age group, educational status, previous use of HM, presence of health problems, drug availability, distance to health facilities (HFs), and attitude towards the use of HMs as candidates for multivariate analysis at *p*-value < 0.25 (Table 3). Accordingly, educational status (*p*-value ≤0.05), previous use of herbal medicines (*p*-value ≤0.05), presence of health problems (*p*-value ≤0.005), drug availability (*p*-value ≤0.001) and distance to the health facilities (*p*-value ≤0.001) were found to have a significant association in multivariate logistic regression analysis (Table 4). The odds of using herbal medicines during pregnancy who can’t read and write were 9.32 times higher than those women who attend more than diploma (AOR: 9.32 95% CI ((2.34, 37.10)). Pregnant women who were with previous experience of using herbal medicines were 3.14 times more likely to use herbal medicine as compared to those who hadn’t previous experience with herbal medicines (AOR: 3.14, 95% CI ((1.38–7.16). Respondents who had health problems were 3.26 times higher than those who hadn’t health problems to use herbal medicines (AOR: 3.26, 95% CI: (1.50–7.09). The odds of using herbal medicines during pregnancy were 6.15 times higher than for those who residing greater than or equal to 5kms from home to the nearest health facility as compared to those women who residing less than 5kms (AOR:6.15, 95% CI: (2.49–15.23). There was also significant association between the use of herbal medicines during pregnancy and drug availability in the health facilities. The odds of using herbal medicine during pregnancy were 9.87 folds higher if drugs were not available as compared to if drugs were available in the health facility (AOR: 9.87, 95% CI: (4.32–22.55).

**Table 3:** Bi-variate logistic analysis result of herbal medicine use during pregnancy in Debre Tabor Town, Ethiopia, 2019

Variable category	HM use		COR(95%CI)	***P*** value
	Yes (n)	No (n)		
Age				
15–19	1	4	1	
20–29	18	72	1(.105–9.501)	1.00
30–39	54	70	.324(.035–2.983)	.32
40–49	22	21	.239(.025–2.313)	.216
Educational status				
Unable to read & write	17	75	7.353(2.760–19.589)	<.001
Can Read & write	18	44	4.074(1.511–10.984)	.006
Primary education	17	19	1.863(0.649–5.345)	.247
Secondary education	28	20	1.190(0.435–3.256)	.734
Diploma and above	15	9	1	
Occupation				
Farmer	7	13	1.238(0.311–4.934)	.762
Self employed	32	72	1.500(0.492–4.569)	.476
Housewife	32	49	1.021(0.331–3.144)	.971
Unemployed	10	14	.933(0.251–3.472)	.918
Student	8	10	.833(0.208–3.345)	.797
Government employed	6	9	1	
Residence				
peri-urban	57	98	.947(0.567–1.582)	.835
Urban	38	69	1	
Prior use of herbal medicines				
Yes	20	78	3.827(1.841–5.866)	< .001
No	75	89	1	
Presence of health problems				
Yes	37	105	2.655(1.581–4.458)	< .001
No	58	62	1	
Did you attend ANC?				
No	44	80	1.066(.643–1.766)	.804
Yes	51	87	1	
Drug availability				
No	32	130	6.917(3.948–12.118)	<.001
Yes	63	37	1	
Number of ANV visits				
≤2	27	46	1.239(.610–2.517)	.553
≥3	24	33	1	
Distance to health facilities				
≥5 km	51	127	2.739(1.600–4.689)	<.001
< 5 km	44	40	1	
Attitude towards the use of herbal medicines				
Positive attitude	34	100	2.678(1.590–4.510)	<.001
Negative attitude	61	67	1	

1 = Reference group

**Table 4:** Multivariate logistic analysis result of herbal medicine use during pregnancy in Debre Tabor Town, Ethiopia, 2019

Variables	HM use		COR[95%CI]	AOR[95%CI]
	Yes(n)	No (n)		
Educational status				
Unable to read & write	17	75	7.353(2.760–19.589)***	5.687(1.572–20.570)*
Read & write	18	44	4.074(1.511–10.984)*	2.438(0.637–9.337)
Primary education	17	19	1.863(0.649–5.345)	1.313(0.324–5.324)
Secondary education	28	20	1.190(0.435–3.256)	0.530(0.138–2.045)
Diploma and above	15	9	1	1
Previous use of HM				
Yes	20	78	3.287(1.841–5.866)***	3.029(1.410–6.510)*
No	75	89	1	1
Presence of health problems				
Yes	37	105	2.655(1.581–4.458)***	2.668(1.304–5.459)**
No	58	62	1	1
Drug availability in the HF				
No	32	130	6.917(3.948–12.118)***	11.584(5.342–25.123)***
Yes	51	87	1	1
Distance to HF				
≥5 km	51	127	2.739(1.600–4.689)***	4.318(1.968–9.473)***
< 5 km	44	40	1	1
Attitude towards the use of HMs				
Positive attitude	34	100	2.678(1.590–4.510)***	2.141(1.025–4.471)
Negative attitude	61	67	1	1

Significant at p-value < 0.05*, P-value ≤0.005**, p-value ≤0.001***

In this study there was no significant association between HM use during pregnancy and age group, residence and respondents’ attitude towards the use of herbal medicines.

### Qualitative findings

The qualitative study was conducted to elicit information about the use of herbal medicine during pregnancy. Focus group was done with one group of 12 pregnant women and 6 individuals for in-depth interviews from FGD. Their ages ranged from 22 to 42 years. Two major themes were emerged in qualitative data analysis. These were reported as facilitators of herbal medicine use and commonly used herbal medicines.

### Facilitators for the use of herbal medicines during pregnancy

Major reasons mentioned by respondents as facilitators were: cultural beliefs to strengthening the pregnancy, previous experience with herbal medicines, distance to modern healthcare, beliefs that herbal medicines are effective in treating many ailments, presence of health problems, and dissatisfaction with modern health service.

### Cultural beliefs to strengthening the pregnancy

The participants belief that once someone is pregnant, she needs herbal medicine to ‘strengthen the pregnancy. By strengthening, the women meant preventing the pregnancy from miscarriage. Herbal medicine played a very central role in the care of pregnancy because it was believed to stabilize the pregnancy during the early period.

As one respondent in the in-depth interview said;

“*I don’t know but others say that during the fourth and half month they used this medicine and they call it strengthened in order to prevent a miscarriage”* (Participant #2)

### Previous experience with herbal medicine use

Some of the respondents in the FGD had previous exposure to herbal medicines. After evaluating the effect of the herbal medicines, they decided to use them again. Because they perceive herbs were natural and safer than conventional medicines.

As one respondent in the in-depth interview noted;

*“I had taken traditional medicines before this time and I have checked its recuperation ability for me so if I got a disease, I will not go to the hospital rather use traditional herbal medicine confidentially”. (*Participant #6)

### Distance to modern healthcare

Focus group participants stated that during pregnancy, especially after the third trimesters, they usually experience weakness or tiredness. Therefore, the distance between their home and health facility had a decisiveness role in using and not using herbal medicines. If the distance between home and health facility was slightly far it was difficult to go to the hospital. So, when they felt some illness, they used herbs from backyards.

A respondent from the in-depth interview had to say:

*“Occasionally, I used cultural medicine when I got sickened. Because the distance between my home and health center is so far, I have faced tiredness while I have gone to the health facility, especially during my pregnant situation. Therefore, I used traditional treatment like leaves”. (*Participant #1)

### Beliefs that herbal medicines are effective in treating many ailments

Most of the participant in the FGD explained that some diseases, such as *yewofbeshita* (Herpes Zoster) were treated by only using herbal medicines.

Regarding this a participant from FGD had to say:

“*The recuperation ability of HM from my sickness over some diseases So I take herbal medicines if I do not relief from my sickness, when I take modern medication” example yewofbeshita”* (Participant #3)

### Presence of health problems

Pregnant women with chronic illnesses were quite high in the use of herbal medicines. Most of the pregnant women in the FGD think that herbal medicines were effective to treat chronic diseases. This was strengthened by one of the participants in the in-depth interview as follows,

*“U knows … . traditional medicines are very important. I use it because I have high blood pressure and physicians order medicine for this disease to use forever throughout my life every day. This is too tedious to take in such ways every day. So I will prefer to take traditional herbal medicines rather than this because I have seen a change when I use it.” (*Participant #5)

### Dissatisfaction with modern medicine use

Some respondents articulated that they were dissatisfied with the result of modern medicine use because health care providers didn’t give them due attention.

Regarding this a respondent in the in-depth interview stated:

*“I am not feeling comfortable in modern medicine once I take it after I have gone to the health center by paying for transportation. I do not feel comfortable in essence, the health professional said that you are ok and simply give Panadol as an analgesic but I was in a series of sick conditions. So as the sickness condition increases, I choose to take herbal medicine, in such a way that I have seen changes within a day” (*Participant #4).

### Herbal medicines commonly used by pregnant women

Participants during focus group discussion indicated the use of different herbal medications to manage some pregnancy related minor ailments, such as nausea and vomiting, abdominal cramp, fever and common cold.

Most of the participants agreed that linseed as the most commonly used herb as it was believed to have a facilitator effect of labor. Herbal medicines used by study participants and most common indications were illustrated in Table 5.

**Table 5:** Commonly used herbal medicines by pregnant women in Debre Tabor Town, Ethiopia, 2019

Common name	Scientific name	Most common indications	Formulations
Ginger	*Zingiber officinale*	Nausea, vomiting and abdominal crump	The dried and crushed ginger are taken as a tea on a daily basis
NechiBahrzaf	*Eucalyptus globulus*	Common cold	Fresh leaves are socked in a hot water for some time and fumigation
Tenaadam	Rutachalepensis	Fever, pneumonia	The Fresh leaves are socked in a tea or a coffee for some time and drunk
Telba/linseed	Linumusitatissimum	To facilitate delivery and peptic ulcer diseases	The seed is mixed with boiling water and drunk
Moringa	Moringastenopetala	Hypertension & diabetic Mellitus	The dried leaves of Moringa are crushed and taken as a tea on a daily basis

## Discussion

The present study determined the prevalence and factors associated with the use of herbal medicines during pregnancy among 262 women in *Debre Tabor* Town.

The finding of this study reported that the prevalence of herbal medicines use among pregnant women is 36.3%. This finding is lower than reports from Zimbabwe (69.9%), Iraq (56.7%) and *Hossana,* Southern Ethiopia (73.1%) [9, 10, 16]. The lower prevalence of HMs use in our study might have been due to the difference in the populations studied, sample size difference, the time of the study and differences in socio-cultural contexts. A study conducted in Zimbabwe reported that Zimbabwean culture and traditions encourage pregnant women to use traditional medicines to either treat pregnancy-related illnesses or to facilitate delivery as they are believed to be safe [16]. However, our finding is higher than the prevalence reported in Kenya (12%) and Northern Uganda (20%) [8, 14]. The possible justification for the difference might be due to differences in accessibility, affordability and socio-cultural context.

This study also found that family/friends were the most frequently cited source of information about the use of herbal medicines and users tended to trust the benefits of use if recommended by close acquaintances. Family and friends represent the social and cultural environment in which pregnant women live and in part influence to their use of herbal medicines during pregnancy which is similar to another finding conducted in Nairobi, Kenya [8]. This could be linked to the very well-constructed social capital values (social support) of the study communities.

We also discovered the degree of disclosure between herbal medicine users and their health care providers. The result was alarming because only 5.3% of pregnant women disclosed herbal medication use with their doctors. More than 90% of the respondents did not discuss use of herbal medicines with health care providers. The reasons for non-disclosures were: doctors/midwives did not ask (40.3%), forget to inform (36%), afraid of doctors/midwives’ response (15.1%) and it was not important to talk (8.6%). This finding was in line with a survey conducted on Iraqi women, who stated that doctors did not ask (50.53%) and afraid of a doctor’s response (5.3%) were perceived hindrances for not reporting [10]. Moreover, the study conducted in *Nekemt*, Ethiopia stated only 14.29% of the women reported to have received health advices from healthcare workers [10]. The lack of communication between the health care providers and pregnant women who are using herbal medicine may have a harmful effect on the mother as well as the fetus. Therefore, health care providers should advise about the harmful effects of taking herbal medicines during ANC visit [17, 18].

The odds of herbal medicine use during pregnancy were 6.15 times higher for women living more than 5kms from the nearest health facility than those who live less than 5kms. This finding was in line with the study conducted in *Gonder*, Ethiopia [13]. Moreover, our qualitative finding supported this result; respondents explained that if the distance between their home and health facility was slightly far it was difficult for them to go to the hospital and they resort to use herbs from the backyard. However, this finding was different from the study in Northern Uganda, which reported that there was not a significant association between distance from the health facilities and the use of herbal medicine during pregnancy [14]. The difference may be due to a variation in transportation access in the two countries. This study also indicated that the use of herbal medicines during pregnancy was 9.87 times higher, if drugs are not availability in the health facility. This finding was in line with the study conducted in Nairobi, Kenya [8]. However, a study conducted in Northern Uganda reported there was no significant association between the use of herbal medicines during pregnancy and the availability of drugs in the health facility (*p*-value = 0.08) [14]**.** This may imply that the unavailability of medicines in the health facility necessitates the use of herbal medicines among pregnant women and their existence has influenced the result significantly.

Pregnant women who were illiterate (cannot read and write) were 9.32 times more likely to use herbal medicine as compared to those who did attend diploma and above. Similar findings were reported from the study conducted in *Gondar* and *Nekemte*, Ethiopia; and Ghana which stated that use of herbal medicines during pregnancy and educational status has significantly related [11, 13, 19]. This may be due to the fact that as they become more educated the rate of herbal drug usage was decreased, because they are likely to know the side effects of herbal medicine usage during pregnancy [20]. This study also found that the odds of using herbal medicines during pregnancy were 3.14 times higher to those participants who had previous experience of herbal medicines use as compared to those who hadn’t prior use. Similarly, a study conducted in Saudi reported that there was a significant association between the use of herbal medicines during pregnancy and prior use [15]. Our qualitative finding also articulated prior use of herbal medicines, by pregnant women, was facilitator of herbal medicines use. After assessing and reassessing the effect of the herbal medicines they decided to use them again. Because they perceived herbs were natural and safer than conventional medicines. The findings also indicated that respondents who had health problems were 3.26 times more likely to use herbal medicines during pregnancy than those who hadn’t health problems. This result was similar to the study conducted in *Gondar*, Ethiopia [13]. This was also augmented by our qualitative finding; which reported that pregnant women with chronic illnesses were quite high in the use of herbal medicines.

Respondents in the FGDs mentioned that *Ginger,Tenaadam*, *Telba*, *NechiBahrzaf*, and *Moringaas* a specific example of herbal medicine use during pregnancy. The pattern of herbal medicine use in our study was almost similar to the study done in *Hosanna* town, southern Ethiopia, where *garlic, ginger, tenaadam*, and *damakasse* were reported to be the commonest herbs used by pregnant women [9] However, a study conducted in Norway reported Echinacea, chamomile, cranberry, and iron-rich herbs as traditional medicines used by pregnant women [21]. The difference in patterns across different countries may be due to differences in accessibility and geographical distribution of herbs. In this study respondents mentioned facilitate labour, pneumonia, nausea and vomiting, abdominal cramp, fever, common cold, hypertension, as a specific example of indications treated with herbal medicines during pregnancy. This finding was similar to the study conducted in Bangladesh [22].

### Strength and limitation of the study

This study was used mixed-method approaches that provide a better understanding of the use of herbal medicines during pregnancy. The principal investigator was native to the study community and this minimizes linguistic and cultural barriers, otherwise, insider bias. Like all self-reported exposure assessments, under reporting is very likely. As it is cross-sectional, it fails to show seasonal variability in the use of herbal medicine Moreover, the study was not able to look at the effectiveness or safety and side effects of the herbal medicines that were mentioned.

## Conclusion

Herbal medicine use during pregnancy was a common experience, and it’s linked to educational status, prior use of herbal medicines, drug availability, presence of health problems and distance to the health facilities. Pregnant women depend mainly on family/ friends as a source of information about herbal medicine use. Ginger (*Zingiber officinale*),Tenaadam (*Ruta chalepensis*), Telba (Linumusitatissimum), NechiBahrzaf *(Eucalyptus globulus)* and Moringa (Moringa stenopetala were the most commonly used herbs among pregnant women, and the most popular indication) and the most common indication for use were pneumonia, nausea and vomiting, abdominal cramp, fever, common cold, and hypertension. Given the high prevalence of herbal medicine and the low rate of disclosure, health care professionals should be willing to explore herbal medicine use with their pregnant patients, since it will result in a better health outcome. Moreover, a detailed study on commonly used herbs to establish the efficacy, safety and side effects of these herbs to ensure the well-being of the mother and foetus would be recommended.

## Supplementary Information

**Additional file 1.**

## Data Availability

The datasets are available from the corresponding author upon reasonable request.

## Abbreviations

ANC: Anti natal care
AOR: Adjusted odds ratio
CI: Confidence interval
OR: Odds ratio
SPSS: Statistical Package for the Social Sciences
WHO: World Health Organization
