# Supplementary material for: Herbal medicines use and associated factors among pregnant women in Debre Tabor town, north West Ethiopia: a mixed method approach
Source: BMC Complement Med Ther. 2021 Oct 26;21:268. doi: 10.1186/s12906-021-03439-3 (PMC8547058; doi:10.1186/s12906-021-03439-3)
Supplement: Supplementary file 1 — Additional file 1. [file 12906_2021_3439_MOESM1_ESM.docx]

**WOLLO UNIVERSITY**

**COLLEGE OF MEDICINE AND HEALTH SCIENCES**

**DEPARTMENT OF PHARMACY**

**Informed consent agreement form, English version**

Title of the thesis project: to assess the prevalence and associated factors of herbal medicine use among pregnant women in *Debre tabor* Town, Northwest Ethiopia.

I am aware that this research undertaking is a postgraduate MSc. a degree research project which is fully supported and coordinated by Wollo University and the designated principal investigator is Getu Tesfaw.

I have been also fully informed in the language I understood and about the research project objective to assess the prevalence and associated factors of traditional medicine use among pregnant women in *Debre tabor* town, Northwest Ethiopia. I have been informed that all the information I shall provide to the interviewer will be kept confidential. I understood that research has no risk and no compensation. I also know that I have the right to withhold information, skip questions to answer or to withdraw from the study at any time. I have been informed that nobody will impose on me to explain the reason for withdrawal. It is also clear that there will be no effect at all in my health benefit or other administrative effects that I get from the district. I have been assured of the right to ask information that is not clear about the research before and/or during the research work by contacting:

1. Wollo University, Office phone: +251 (0) 331190588

2. Principal investigator name and address: Getu Tesfaw, cell Phone:

+251 (0) 941567054

3. Supervisor name and address: _____________________

I have read this form, or it has been read to me in the language I comprehend, and I understood the condition stated above; therefore, I am willing and confirm my participation by signing this consent form. Pregnant women to participate in the study: (Mark one of them for verbal/oral consent)

Yes No

Name of interviewer_________________ Signature __________Date ____________

## English Questionnaire

Participant ID No___________ Kebele ____ Interviewer name_________________________ Date of interview____________ Starting time _____________Ending time ___________ please encircle the correct answer

1. Age in years ---------------------
2. Marital status
3. Single
4. Married
5. Divorced
6. Widowed
7. Religion
8. Orthodox
9. Muslim
10. Protestant
11. Others
12. Educational status
13. Unable to read & write
14. Read and write
15. primary school
16. secondary school
17. diploma and above
18. Occupation
19. farmer
20. government-employed
21. self-employed
22. student
23. unemployed
24. housewife
25. Residence
26. Peri-urban
27. urban
28. Have you ever used HM prior to this pregnancy
29. Yes
30. No
31. Do you use any herbal medicine during current pregnancy?
32. Yes (continue to Q 9)
33. No (skip to Q 17)
34. If your answer Q No.8 is yes, in which period of pregnancy?
35. 1st trimester (0-3 months)
36. 2nd trimester (4-6 months)
37. 3rd trimester (7- 9 months)
38. Throughoutpregnancy
39. Who recommended you to take the herbal medicine?
40. No one (myself)
41. Recommendation from family/friends
42. Recommendation from health professionals
43. Recommendation from herbalists’
44. How often do you use herbal medicine?
45. daily
46. weekly
47. monthly
48. When sick
49. Where do you obtain herbal medicine?
50. From the forest/backyard
51. From traditional herbalists
52. From shop
53. From pharmacies
54. Other (specify)
55. What are your reasons for using herbal medicines? [ more than one answer is possible]
56. Beliefs ineffectiveness of herbal medicines 1. Yes 2. No
57. They are safe to use during pregnancy 1. Yes 2. No
58. It is part of our culture to use it 1. Yes 2. No
59. It is always available when I need them 1. Yes 2. No
60. To prevent miscarriages 1. Yes 2. No
61. Other (specify)……………
62. Have you had any side effects in your use of herbal medicine?
63. Yes
64. No
65. If yes, what side effects/complications have you experienced since you began using herbal medicine?(more than one response is possible)
66. Vomiting 1. Yes 2. No
67. Abortion 1. Yes 2. No
68. Headache 1. Yes 2. No
69. Rashes 1. Yes 2. No
70. Diarrhea 1. Yes 2. No
71. Heartburn 1. Yes 2. No
72. other
73. If your answer Q No.8 is No, why do you not use herbal medicines during your pregnancy? [more than one response is possible]
74. The side effects could be dangerous 1. Yes 2. No
75. It is not safe for pregnant women 1. Yes 2. No
76. I do not believe in the effectiveness of herbal medicines 1. Yes 2. No
77. It is not properly processed 1. Yes 2. No
78. Friends/family have advised me not to use it 1. Yes 2. No
79. Health professionals have advised me not to use it 1. Yes 2. No
80. Others ………………………………
81. Number of parity------------------------
82. Concurrence disease: Did you have any pregnancy-related illnesses or diseases during the recent pregnancy?
83. Yes
84. No
85. If Yes, for Q No18, what type of disease do you have? (more than one answer is possible)
86. Hypertension 1. Yes 2. No
87. Asthma 1. Yes 2. No
88. Diabetes Mellitus 1. Yes 2. No
89. Epilepsy 1. Yes 2. No
90. Other (Please specify)--------------------------------
91. Did you attend ANC?
92. Yes
93. No
94. If yes for Q No 20, how many times did you attend antenatal care during your most recent pregnancy? ----------------------------
95. Would you tell your HCPs about HM use during the antenatal visits?
96. Yes (skip to Q 24)
97. No (continue to Q 23)
98. If No, Why?
99. Forget to inform
100. Doctor/midwife didn’t ask
101. Afraid of doctors or midwives response
102. It was not important to disclose/talk
103. If yes, what was your doctor’s advice regarding herbal medicine use?
104. Use of herbal medicine is harmful
105. There are side effects associated with herbal medicine use
106. There are side effects associated with the use of both HM and modern medicine together
107. Discontinue use of herbal medicine
108. How far is your home from the conventional health facility in Km? ----------

1. What is the main transport modality to the nearest hospital?
2. On Foot
3. By Bajaj
4. By Ambulance
5. by Taxi
6. Other(specify)
7. Drugs availability in health facilities
8. Available
9. Not available

**Attitude of the Respondents Towards the use of herbal medicines in Debre Tabor Town**

Please indicate whether you Strongly Agree, Agree, Not sure, Disagree, or strongly dis-agree about the following statements

| Statement | Strongly Agree | Agree | Not sure | Disagree | Strongly Dis-agree |
| --- | --- | --- | --- | --- | --- |
| 1. Herbal medicine is more safer for me and my baby during pregnancy than modern medicines |  |  |  |  |  |
| 1. There are illnesses or conditions for which herbal medicine is more effective than Western medicine |  |  |  |  |  |
| 1. Herbal medicine is more accessible than conventional medicine |  |  |  |  |  |
| 1. Herbals are beneficial recommended by health professionals |  |  |  |  |  |
| 1. Herbals are beneficial if recommended by family/friends |  |  |  |  |  |
| 1. Herbals are beneficial if recommended by herbalists |  |  |  |  |  |
| 1. Prefer first to visit herbal medicine practitioner than modern medicine |  |  |  |  |  |

Thank you very much for your cooperation in answering to the questions above.
